# The Sanatory Condition of the Laboring Population of New York, with Suggestions for Its Improvement; a Discourse (with Additions) Delivered on the 30th of December, 1844, at the Repository of the American Institute

**Published:** 1845-04

**Authors:** 


*The Sanatory condition of the Laboring population of New York, with suggestions for its improvement; a Discourse (with additions) delivered on the 30th of December, 1844, at the Repository of the American Institute.* By JOHN H. GRISCOM, M. D., Fellow of the College of Physicians and Surgeons; Physician of the New York Hospital; late Physician of the City and Eastern Dispensaries. 8vo. pp. 58. Harper & Brothers—New York, 1845.

By his connection with the various public institutions in the city of New York, and particularly from the opportunities afforded him while acting as City Inspector, the author of this publication has had unusual means of acquiring correct information on the subjects of which he has written; and the facts which he has narrated, and the suggestions he has made in reference to the circumstances, are such as should claim the early and serious attention of the inhabitants and constituted authorities of that growing and populous place. Health ought to be the first consideration, with communities as well as individuals, and that blessing can hardly be expected where there is a total neglect of the means necessary to preserve it. The cities in the United States are yet comparatively small, and their inhabitants have not hitherto experienced the evils of over crowding, but the time is rapidly approaching when, it is to be apprehended, they will be aroused to the consequences of their present negligence. Our author justly remarks that—"no duty can engage the attention of the magistracy of a city or state, more dignified in itself, more beneficial to the present generation, or more likely to prove useful to their descendants, than that of procuring and maintaining a sound state of the public health." Our space will not allow us to copy many passages which we had marked when reading this interesting pamphlet, but the following account of the system of tenantry practised in our sister city, is so graphic, and shows a custom so different from what prevails in Philadelphia, that we cannot refrain from extracting it.

"The basis of these evils," observes Dr. Griscom, "is the subjection of the tenantry to the merciless inflictions and extortions of the *sub-landlord.* A house, or a row, or court of houses, is hired by some person of the owner, on a lease of several years, for a sum which will yield a fair interest on the cost. The *owner* is thus relieved of the great trouble incident to the changes of tenants, and the collection of rents. His income is sure from one individual, and obtained without annoyance or oppression on his part. It then becomes the object of the lessee, to make and save as much as possible, with his adventure, sufficient sometimes to enable him to purchase the property in a short time.

"The tenements, in order to admit a greater number of families, are divided into small apartments, as numerous as decency will admit. Regard to comfort, convenience, and health, is the last motive; indeed, the great ignorance of this class of speculators (who are frequently foreigners and keep a grog shop on the premises) would prevent a proper observance of these, had they the desire. These closets, for they deserve no other name, are then rented to the poor, from week to week, or month to month, the rent being almost invariably required in advance, at least for the first few terms. The families moving in first, after the house is built, find it clean, but the lessee has no supervision over their habits, and however filthy the tenement may become, he cares not, so that he receives his rent. He and his family are often found steeped as low in depravity and discomforts as any of his tenants, being above them only in the possession of money, and doubtless often beneath them in moral worth and sensibility.

"It is very frequently the case that families, after occupying rooms a few weeks, will change their location, leaving behind them all the dirt which their residence has occasioned. Upon this the next comer will sit down, being so much occupied with the hurry of moving, and with the necessity of placing their furniture immediately in order, that attention to cleansing the apartment is out of the question, until they are 'settled,' and then, if done at all, it is in the most careless and inefficient manner. Very often, perhaps in a majority of cases in the class of which I now speak, no cleaning other than washing the floor is ever attempted, and that but seldom. Whitewashing, cleansing of the furniture, of bedding, or persons, in many cases is *never* attempted. Some have old pieces of carpet, which are never shaken (they wouldnot bear it), and are used to hide the filth on the floor. Every corner of the room, of the cupboards, and the entries and stairways, is piled up with dirt. The walls and ceilings, with the plaster broken off in many places, exposing the lath and beams, and leaving openings for the escape from within of the effluvia of vermin, dead and alive, are smeared with the blood of unmentionable insects, and dirt of all indiscribable colors. The low rooms are diminished in their areas by the necessary encroachments of the roof, or the stairs leading to the rooms above; and behind and under them is a hole, into which the light of day never enters, and where a small bed is often pushed in, upon which the luckless and degraded tenants pass their nights, weary and comfortless.

"In these places, the filth is allowed to accumulate to an extent almost incredible. Hiring their rooms for a short period only, it is very common to find the poor tenants moving from place to place, every few weeks. By this practice they avoid the trouble of cleansing their rooms, as they can leave behind them the dirt which they have made. The same room being occupied in rapid succession by tenant after tenant, it will easily be seen how the walls and windows will become broken, the doors and floors become injured, the chimneys filled with soot, the whole premises populated thickly with vermin, the stairways, the common passage of several families, the receptacle for all things noxious, and whatever of self-respect the family might have had, be crushed under the pressure of the degrading circumstances by which they are surrounded."

We commend this publication to the serious perusal of all Philanthropists and Legislators, and especially those whose duty it is to guard the health of our large cities. It is evidently the production of an intelligent and observing mind, influenced by benevolent motives.

